# The Protective Effect of Gangliosides on Lead (Pb)-Induced Neurotoxicity Is Mediated by Autophagic Pathways

**DOI:** 10.3390/ijerph13040365

**Published:** 2016-03-25

**Authors:** Hongtao Meng, Lan Wang, Junhong He, Zhufeng Wang

**Affiliations:** Department of Neurology, Shanxi Hospital of the Armed Police Force, Xi’an 710054, China; linshasn@163.com (L.W.); sphm1987@fmmu.edu.cn (J.H.); sp1665@sohu.com (Z.W.)

**Keywords:** gangliosides, lead, apoptosis, autophagy

## Abstract

Lead (Pb) is a ubiquitous environmental and industrial pollutant and can affect intelligence development and the learning ability and memory of children. Therefore, necessary measures should be taken to protect the central nervous system (CNS) from Pb toxicity. Gangliosides are sialic acid-containing glycosphingolipids that are constituents of mammalian cell membranes and are more abundantly expressed in the CNS. Studies have shown that gangliosides constitute a useful tool in the attempt to promote functional recovery of CNS and can reverse Pb-induced impairments of synaptic plasticity in rats. However, the detailed mechanisms have yet to be fully understood. In our present study, we tried to investigate the role of gangliosides in Pb-induced injury in hippocampus neurons and to further confirm the detailed mechanism. Our results show that Pb-induced injuries in the spatial reference memory were associated with a reduction of cell viability and cell apoptosis, and treatment with gangliosides markedly ameliorated the Pb-induced injury by inhibition of apoptosis action. Gangliosides further attenuated Pb-induced the abnormal autophagic process by regulation of mTOR pathways. In summary, our study establishes the efficacy of gangliosides as neuroprotective agents and provides a strong rationale for further studies on the underlying mechanisms of their neuroprotective functions.

## 1. Introduction

Lead (Pb), as a ubiquitous environmental toxicant, remains a major health problem [1,2]. Severe and acute Pb poisoning can cause encephalopathy, convulsion, coma and death. Exposure to Pb may cause a broad spectrum of toxic effects in both adults and children [3]. Although China has made significant strides in controlling Pb pollution, more efforts are warranted to reduce Pb poisoning. Recently, the toxic effects of Pb on the central nervous system (CNS) have gained increased attention [4,5]. Previous studies showed that Pb affected both the release and reuptake of several neurotransmitters controlled by voltage-gated Ca^2+^ channels [6]. Pb also caused tau hyper-phosphorylation and α-synuclein accumulation, leading to the enhancement of type I (apoptosis) and type II programmed cell death (autophagy) in the hippocampus [7]. Pb could also cause injuries in CNS probably via disrupting function of the brain barriers, including blood brain barrier [8] and blood-cerebrospinal fluid barrier [9]. Therefore, much work should be conducted to protect the CNS from Pb toxicity.

Gangliosides are sialic acid-containing glycosphingolipids (GSLs) ubiquitously distributed in tissues and body fluids, and more abundantly expressed in the nervous system [10]. Accumulated evidence suggested that gangliosides play an important role in many neuron functions, such as proliferation, neurogenesis, receptor transmission and so on [11,12,13]. In the central nervous system (CNS) of higher vertebrates, 80%–90% of gangliosides are the so-called GM1, GD1a, GD1b and GT1b components. As the hippocampus has various cell types and plays an important role in learning and memory, it has been a particularly popular site for studies of gangliosides distribution and function [14]. Studies have shown that exogenously administered gangliosides can provoke CNS regeneration and prevent CNS degeneration following trauma. Gangliosides can also prevent or mitigate the progression of neurodegenerative diseases, including Alzheimer’s disease (AD) [15] and Parkinson’s disease (PD) [16,17,18]. Previous studies have shown that gangliosides could reverse lead-induced impairments of synaptic plasticity in rats [19]. However, the detailed mechanism behind this activity is still unclear.

The aim of our research was to investigate the role of gangliosides in Pb-induced injury in hippocampus neurons and to further confirm the details of the corresponding mechanism of action. Our study showed that gangliosides protect hippocampus neurons from Pb-induced neurotoxicity by inhibition of cell apoptosis and attenuate Pb-induced abnormal autophagic processes by regulation of mTOR pathways. These results of our research shed new light on an achievable solution to neuron injuries induced by lead exposure.

## 2. Materials and Methods

### 2.1. Chemicals and Reagents

Gangliosides mixture (GMIX; 18% GM1, 55% GD1a, 15% GD1b, 10% GT1b, and 2% others), 3-methyladenine (3-MA) and methylthiazolyltetrazolium (MTT), were purchased from Sigma-Aldrich (St. Louis, MO, USA). Dulbecco’s modified Eagle’s medium (DMEM), and fetal bovine serum were purchased from Gibco (New York, NY, USA). HRP-conjugated goat anti-rabbit IgG was purchased from Abcam (Cambridge, MA, USA). Rabbit anti-Beclin-1, LC3, caspase-3, PARP, p-mTOR, t-mTOR, p-70s6k, t-70s6k, Atg5 and β-actin monoclonal antibodies were purchased from Cell Signal Technology (Danvers, MA, USA).

### 2.2. Animals and Treatments

Adult male and female Sprague-Dawley rats (180–240 g) were purchased from the animal center of the Fourth Military Medical University (Xi’an, China). The animals were housed in stainless-steel cages in a temperature-controlled (22–24 °C), 12/12 light/dark room, and were given free access to food and drink water.

To establish a Pb exposure *in vivo* model, adult virgin females were placed into the cage of a stud male (two females per each male), until they mated as indicated by the presence of a vaginal plug. After mating, pregnant females were randomly divided into four groups: Control group (*n* = 6) and GMIX group (*n* = 7), which received deionized water; Pb group (*n* = 7) and Pb+GMIX group (*n* = 7), which received water with 300 ppm lead acetate after the birth of pups (day 0). The infant rats in Pb group and Pb+GMIX group received lead from milk before their weaning. After weaning, the pups in control group and Pb group were injected intraperitoneally with saline (0.2 mg/100 g) for 14 days, and those in GMIX group and Pb+GMIX group were injected intraperitoneally with GMIX (0.2 mg/100 g) for 14 days.

At the end of every week during exposure, blood samples (40 μL) was collected from the tail vein of each rat and the concentration of Pb was measured in duplicate using graphic furnace atomic absorption spectrometry. At the end of the abovementioned experiments, rats were executed and the hippocampus tissues were isolated for immunohistochemistry and western blot assays. Animal-elated experimental procedures were performed according to the Guidelines for Animal Experimentation of Fourth Military Medical University, with the approval of the Institutional Animal Care and Use Committee (D1408L06719).

### 2.3. Morris Water Maze

All the rats in each group were tested. The behaviors of the rats (latency period, path length, swim speed, and navigation path) were monitored by a video camera mounted on the ceiling above the center of the pool, and rats in each group finished four trials in one day and continued for 5 days. A trial began with placing a rat in the water facing the wall of the pool at one of the starting point. If the rat failed to find the platform within 120 s, it was manually guided to the platform. After the last trial, animals were carefully dried off and returned to their home cages. 24 h after the hidden platform test, the escape platform was removed, and the same rats were allowed to swim freely for 120 s.

### 2.4. Cell Culture

The HT22 hippocampal nerve cell lines were purchased from the American Type Culture Collection (ATCC, Manassas, VA, USA) and maintained in DMEM medium, supplemented with 10% heat-inactivated fetal bovine serum, 100 units/mL of penicillin, and 100 mg/mL of streptomycin in a water-saturated atmosphere of 5% CO_2_ at 37 °C. In all experiments, exponentially growing cells were used.

### 2.5. MTT Assay

Cell viability was assessed using the MTT assay. Briefly, cells were seeded on 96-well plates at a density of 5 × 10^3^ cells/well. Once confluent, cells were treated for 48 h with graded concentrations of lead acetate (1, 10, 20, 50 and 100 μM) or different concentration of GMIX (10, 30, 50 and 100 μg/mL) to determine cell viability. Next, the cells were treated with 0.5 mg/mL MTT (dissolved in PBS and filtered through a 0.2-mm membrane) at 37 °C. Four hours later, the formazan crystals were dissolved in DMSO, and the absorption values were determined at 492 nm on an automated Infinite^®^ 200 microplate reader (Tecan: Mannedorf, Switzerland).

### 2.6. TUNEL Assay

The detection of apoptosis was performed with the *in situ* TUNEL method. The procedure was conducted according to the manufacturer’s (Roche: Berlin, Germany) protocol. For animal experiments, rats were anesthetized with 2% sodium amytal and perfused with 0.9% saline, followed by 4% paraformaldehyde. Brains were taken and dehydrated in 30% sucrose in phosphate buffered saline (PBS, pH 7.4) and then were cut longitudinally into 20 μm sections. Brain sections were performed by NeuN at 4 °C for overnight. Next, sections were rinsed three times with PBS, labeled at 37 °C for 2 h with the TUNEL reaction mixture, rinsed again with PBS, followed by DAPI staining at 37 °C in darkness for 25 min. The number of apoptotic cell was counted from 10 to 20 random fields (200×) of a coverslip. Cells were examined and recorded under an Olympus BX51 fluorescence microscope equipped with DP-BSW software (Olympus: Tokyo, Japan).

### 2.7. Flow Cytometric Analysis

HT22 cells were cultured in medium containing 10 μM Pb, 30 μg/mL GMIX or 10 μM Pb+ 30 μg/mL GMIX. After treatment for 48 h, cells were washed twice with 0.01 M PBS and suspended in 200 μL binding buffer. Next, cells were incubated with 10 μL Annexin V-FITC and 5 μL PI for 30 min at 4 °C in dark room and then assessed for cell apoptosis by flow cytometry (Beckman Coulter: Brea, CA, USA). Each experiment was performed in triplicate.

### 2.8. Western Blot

At the end of each treatment, cell lysates were prepared by incubation on ice with lysis buffer (50 mM Tris-HCl (pH 7.5), 20 mM NaCl, 5 mM EDTA, 1% TX-100, 0.1% SDS, 5% glycerol + protease inhibitors), and centrifuged at 20,000 *g*. The supernatant was collected and protein concentration was determined using the Pierce BCA Protein Assay Kit (Thermo: Tokyo, Japan). Equal amounts of sample were loaded on SDS-PAGE and transferred to nitrocellulose membranes at 250 mA for 1 h. Membranes were stained with 0.5% Ponceau in 1% acetic acid for confirmation and were blocked for 2 h in TBST (10 mM Tris-HCl, pH 7.4, 150 mM NaCl, 0.1% Tween-20) containing 5% fat-free dried milk, and then incubated with the primary antibodies overnight. Membranes were incubated with corresponding HRP-labeled secondary antibodies and β-actin was selected as an internal control. Protein bands were visualized by chemiluminescence with the enhanced chemiluminescent detection kit according to the manufacturer’s instructions.

### 2.9. Statistical Analysis

All experiments were performed at least three times, and results were expressed as means ± SD. The results were analyzed by one-way ANOVA followed by a SNK-q test for multiple comparisons. All analyses were performed using the Statistical Package for the Social Sciences (SPSS) software (SPSS Inc.: Chicago, IL, USA). Data were considered statistically significant for *p* < 0.05. More specific indices of statistical significance were indicated in individual figure legends.

## 3. Results

### 3.1. Gangliosides Mitigate Pb-Induced Injuries to Rats’ Spatial Reference Memory

**Figure 1:**
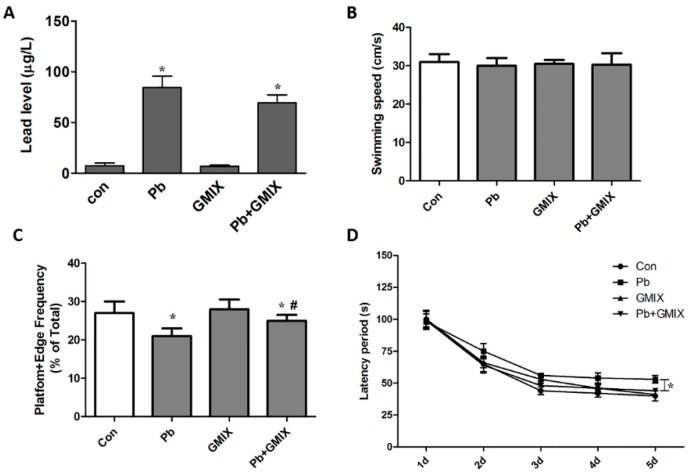
Gangliosides attenuates Pb-induced injuries in spatial reference memory. (**A**) The blood lead levels (BLLs) in four groups of rats; (**B**) Swimming speed in the water maze on the first day of the training; (**C**,**D**) The percentage of time spent in the target quadrant, as indicated by the percentage of time swimming across the area or the edge of the target quadrant to the four quarters. The data are expressed as means ± SD. * *p* < 0.05 *vs.* control group; # *p* < 0.05 *vs.* Pb group.

By establishing an *in vivo* Pb exposure model, we found that treatment with gangliosides after Pb exposure could effectively alleviate Pb-caused neurotoxicity. The blood lead level (BLL) results showed that after Pb exposure for 5 weeks, the BLL in the Pb group and Pb+GMIX group were much higher compared with control and GMIX group (Figure 1A). However, no big difference was noted between the Pb group and Pb+GMIX group, indicating that GMIX treatment couldn’t increase the elimination of Pb. Next, we used a Morris water maze task test to examine the condition of their spatial reference memory. As shown in Figure 1B,C, there were no obvious difference on their swimming speed, which suggested that Pb treatment or GMIX treatment didn’t change rats’ motor ability. From the Pb group results, we found that Pb exposure dramatically affected their spatial reference memory ability, and the percentage of time spent in the target quadrant (platform + platform edge) in the Pb group was much lower than that in control group (21.3 ± 1.5 *vs.* 28.1 ± 1.8, *p* < 0.05). GMIX treatment alone had no notable effect on their spatial reference memory performance, but GMIX administration after Pb exposure attenuated the degree of Pb-induced decrease in spatial reference memory performance (21.3 ± 1.5 *vs.* 25.4 ± 1.6, *p* < 0.05) (Figure 1C). Furthermore, our data showed that Pb exposure also increased the escape latency (54.3 ± 1.1 s *vs.* 44.7 ± 0.9 s, *p* < 0.05), but treatment with GMIX effectively decreased latency period compared with Pb exposure (51.0 ± 1.2 s *vs.* 44.7 ± 0.9 s, *p* < 0.05) (Figure 1D). Taken together, our results indicated that GMIX intervention alleviated Pb-induced decline in the spatial reference memory.

### 3.2. Gangliosides Inhibit Pb-Induced Neural Injuries by Inhibiting Neuron Apoptosis

**Figure 2:**
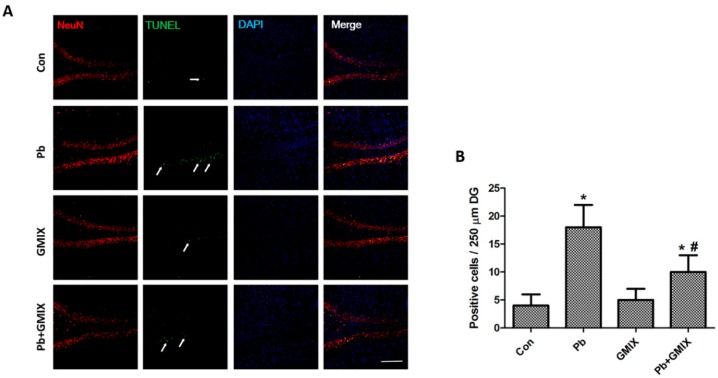
Gangliosides inhibit the number of TUNEL-positive cells in the hippocampus DG region. (**A**) Cell apoptosis is detected by *in situ* NeuN (red), TUNEL (green) and DAPI (blue), and the arrows represent TUNEL positive cells; (**B**) Quantification is performed by counting the number of surviving neurons and apoptotic-like neurons per 250 μm length in the medial DG pyramidal cell layer. The results are expressed as the mean ± SD (*n* = 4, scale bar = 50 μm). * *p* < 0.05 *vs.* control group; # *p* < 0.05 *vs.* Pb group.

To investigate the mechanism of action of gangliosides on Pb exposure-induced neural injuries, TUNEL staining was used to assess the apoptosis in hippocampal dentate gyrus (DG) area. As shown in Figure 2A, Pb exposure induced an obvious increase in TUNEL positive cells in DG areas compared with control group, and GMIX treatment with Pb exposure strongly decreased the number of Pb-induced neural cell deaths in the hippocampal DG region (Figure 2B, *p* < 0.05). These data demonstrate that gangliosides can protect hippocampus neurons from Pb assault by inhibition of cell apoptosis.

### 3.3. Gangliosides Show Cytoprotective Effects Against Pb-Induced Cytotoxicity in HT22 Cells

**Figure 3:**
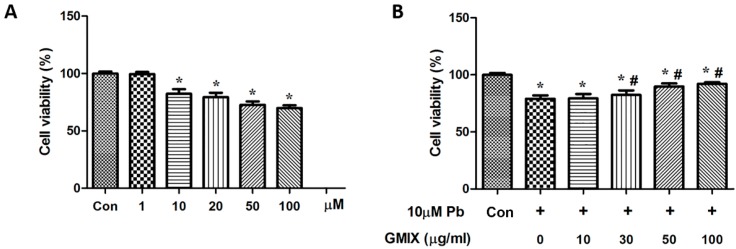
Gangliosides decrease Pb-induced cytotoxicity in HT22 cells. (**A**) Cell viability of HT22 cells after treatment with Pb; (**B**) cell viability of HT22 cells after treatment with GMIX and Pb. The data are expressed as means ± SD of three independent experiments. * *p* < 0.05 *vs.* control group; # *p* < 0.05 *vs.* Pb group.

To further establish the role of GMIX in Pb-induced neurotoxicity, we used HT22 cell for *in vitro* studies. As shown in Figure 3A, cell viability dose-dependently decreased after treatment with control, and ≥10 μM Pb could significantly reduce cell viability (*p* < 0.05). To test the protective role of GMIX, a concentration of 10 μM Pb was chosen. HT22 cells were pre-incubated with GMIX (10, 30, 50, 100 μg/mL) concentrations for 2 h followed by additional 48 h incubation with Pb. As shown in Figure 3B, GMIX alone had no effect on cell viability of HT22 cells, but GMIX inhibited the Pb-induced cytotoxic effect, and 50 μg/mL GMIX pre-treatment markedly increased cell viability in the Pb-treated HT22 cells (*p* < 0.05). Our data reveal that GMIX exerts cytoprotective functions against Pb-induced neural injuries in HT22 cells.

### 3.4. Gangliosides Inhibit Pb-Induced Apoptosis by Altering Protein Expression of Apoptosis-Related Proteins in HT22 Cells

**Figure 4:**
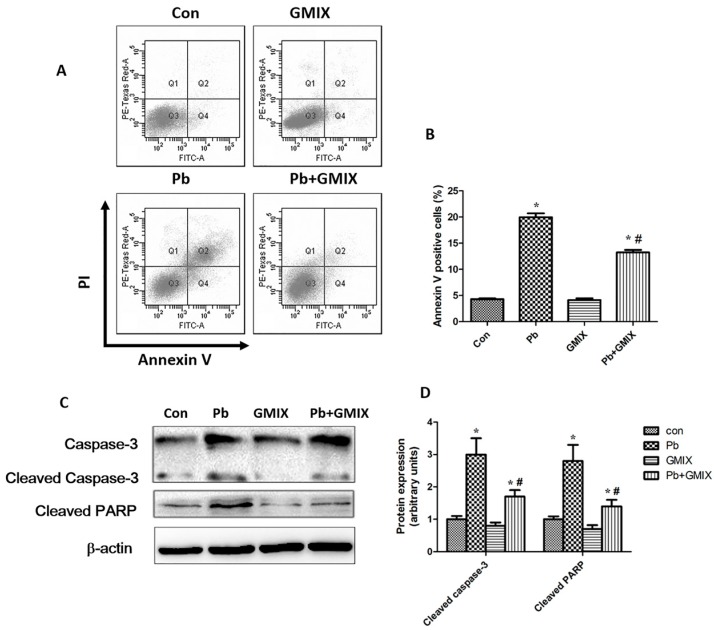
Gangliosides mitigate Pb-induced apoptosis by mediating apoptosis-related proteins in HT22 cells. (**A**) Flow cytometry analysis in response to Con, Pb, GMIX and Pb+GMIX group, and representative dot-plots illustrating apoptotic status were shown; (**B**) Histograms from cell apoptosis were shown for analyzed cells; (**C**) Protein expression of caspase-3 and PARP; (**D**) The values from the densitometry are normalized to the level of β-actin protein and expressed as mean-fold increase. The data are expressed as means ± SD of three independent experiments. * *p* < 0.05 *vs.* control group; # *p* < 0.05, *vs.* Pb group.

To determine whether the Pb-induced reduction of cell viability was associated with apoptosis in HT22 cells, flow cytometry was used. The results showed that Pb increased cell apoptosis in HT22 cells (Figure 4A,B). As shown in Figure 4B, 10 μM Pb treatment caused a ~18.9% population of HT22 cell apoptosis compared with ~4.7% population in the control group (*p* < 0.05), and 50 μg/mL GMIX alone had no significant effect on the ratio of cell apoptosis (Figure 4B). However, pretreatment with 50 μg/mL GMIX after Pb exposure could notably reduce the population of apoptopic cells. These data demonstrate that GMIX protect HT22 cells from Pb toxicity by inhibition of cell apoptosis.

To determine the mechanism of Pb-induced apoptosis, we examined the expression of apoptotic protein caspase-3 and its downstream molecule poly-ADP-ribose polymerase (PARP). The results showed that 10 Pb μM treatment activated caspase-3 and PARP (Figure 4C,D) and increased the cleavage of caspase-3 and PARP. But GMIX pretreatment significantly reduced the degree of cleaved caspase-3 and PARP. Taken together, the results implied that GMIX inhibited Pb-induced neurons apoptosis by inhibiting cell apoptosis.

### 3.5. Gangliosides Inhibit Pb-Caused Autophagy in HT22 Cells

**Figure 5:**
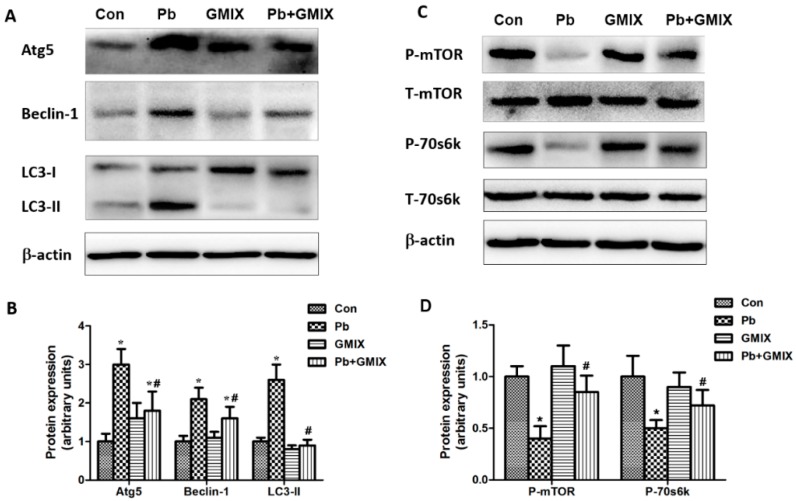
Effect of GMIX on the expression of autophagic related proteins and mTOR pathways in HT22 cells. (**A**) Protein expression of Atg5, Beclin-1 and LC3 in HT22 cells; (**B**) the values from densitometry of Atg5, Beclin-1 and LC3-II are normalized to the level of β-actin protein and expressed as fold increase; (**C**) rhe phosphorylation of mTOR and p70s6k; (**D**) the values from the densitometry of phosphorylated mTOR/p70s6k are normalized to the level of total mTOR/p70s6k. The data are expressed as means ± SD of three independent experiments. * *p* < 0.05 *vs.* control group; # *p* < 0.05 *vs.* Pb group.

As previous studies showed that autophagy played a critical role in hippocampus neurons [7], we therefore investigated whether GMIX could influence autophagy in HT22 cells. We measured the related autophagic proteins Atg5, Beclin-1, LC3 in HT22 cells. Our results showed that 10 μM Pb up-regulated the expression of Atg5, Beclin-1 and enhanced LC3-I to LC3-II conversion (Figure 5A,B). In the Pb+ GMIX group, the protein expression of Atg5 and Beclin-1 were much less than in the Pb group, but higher than in the control group. Notably, GMIX almost completely inhibited Pb-induced LC3-I to LC3-II conversion. The protein levels of Atg5, LC3-II and Beclin-1in GMIX group were almost the same with those in control group.

It is well established that mTOR kinase and its downstream target p70s6k serve as a key signaling molecule in the suppression of autophagy [20], so we next investigated the protein expression of mTOR and p70s6k. Figure 5C,D show that Pb effectively inhibited the phosphorylation of mTOR and p70s6k, and GMIX pretreatment reversed the Pb-induced decreasing in mTOR and p70s6k phosphorylation.

## 4. Discussion

In recent years, great attention has been paid to deciphering the mechanisms and prevention of Pb-induced neurotoxicity [21,22]. Gangliosides have been reported to exert beneficial effects upon the central nervous system and can inhibit neuron injuries after Pb exposure [19]. However, the detailed mechanism has not been clarified yet. Accordingly, the present study embarked on a detailed characterization on the role of gangliosides in Pb-induced neural toxicity *in vivo* and *in vitro*.

Because Pb toxicity mainly impairs spatial reference memory, we firstly established an animal model to verify the effect of gangliosides *in vivo*. As shown in Figure 1, the BLL results showed that gangliosides couldn’t decrease the concentration of Pb in blood, suggesting that gangliosides did not affect the absorption or elimination of Pb. However, the behavioral experiment data suggested that treatment with GMIX effectively diminished Pb-induced cognitive function impairment. These data indicated that gangliosides exerted their protective action not by decreasing the concentration of Pb in our body, but by inhibiting the toxicity of Pb. Previous studies showed that exogenous gangliosides enhanced the learning ability and memory retention in rats at different ages [23]. In our studies, administration of gangliosides alone led to a slight improvement in spatial reference memory (Figure 1), and this may be because the concentration of gangliosides used in our experiment is relatively low.

Previous studies showed that monosialoanglioside prevented acute Pb-induced impairments of synaptic plasticity and inhibited Pb-induced oxidative stress in the hippocampus [24]. However, whether gangliosides could affect the survival of hippocampus neurons is not clear. Therefore, we studied the efficacy of gangliosides in attenuating Pb-induced detrimental effects. By using the TUNEL assay, we established that Pb increased apoptotic cell death in hippocampus DG region, and gangliosides effectively reversed this effect (Figure 2). To further confirm the effect of gangliosides, we used the HT22 cell line to conduct *in vitro* experiments. The results of a MTT assay (Figure 3) and flow cytometric analysis (Figure 4A) *in vitro* showed good consistence with the TUNEL results *in vivo*. To explore the putative pathway of Pb-induced cell apoptosis, we analyzed caspase-3 and its downstream PARP protein expression. Figure 4C shows that Pb increased the cleavage of caspase-3 and PARP, and pretreatment with gangliosides partly reversed these Pb-induced effects. These data further confirmed that the protective effect of gangliosides was mediated by an apoptosis inhibition process.

Autophagy is a general term for pathways by which cytoplasmic materials, including soluble macromolecules and organelles, are delivered to lysosomes for degradation [25]. Recently, much attention has been paid to the role of autophagy in CNS and inflammation [26,27]. Gangliosides have been reported to participate in neural autophagic action. Gangliosides could induce autophagic cell death and increased the formation of autophagic vacuoles as revealed by monodansylcadaverine staining in astrocytes [28]. The NF-kappaB pathway takes part in gangliosides-induced autophagic cell death and activation of astrocytes and inhibition of IKK/NF-kappaB attenuated autophagy of astrocytes [29]. Meanwhile, gangliosidosis, an autosomal recessive lysosomal lipid storage disorder, activated autophagy and led to mitochondrial dysfunction [30]. Although normal autophagy can protect neurons through regulating the environment within the cell, the occurrence of excessive autophagy can lead to autophagic cell death, also known as programmed cell death II. Previous studies have shown that Pb exposure induced excessive autophagy in the hippocampus and increased autophagy-related proteins expression [7]. In our experiments, gangliosides could inhibit Pb-induced up-regulation of Atg5 and Beclin-1, and attenuate Pb-caused LC3-I conversion to LC3-II. What’s more, the phosphorylation of mTOR and its substrate p70s6k also indicated that gangliosides could inhibit Pb-induced abnormal autophagic processes. These results suggested that gangliosides pretreatment exerts its beneficial effects by inhibiting autophagy activation induced by Pb.

## 5. Conclusions

In summary, we have investigated the molecular mechanisms of Pb-induced neurotoxicity and the protective effect of gangliosides on Pb-induced neural damage. Our study establishes the efficacy of gangliosides as neuroprotective agents and provides a strong rationale for further studies on the underlying mechanisms of neuroprotective functions.

## Abbreviations

The following abbreviations are used in this manuscript:

AD	Alzheimer’s disease
BLL	Blood lead level
Pb	Lead
CNS	central nervous system
DG	dentate gyrus
GSLs	glycosphingolipids
PD	Parkinson’s disease
